# Isolation and characterization of Bradykinin potentiating peptides from *Agkistrodon bilineatus* venom

**DOI:** 10.1186/s12953-016-0090-0

**Published:** 2016-01-14

**Authors:** Aisha Munawar, Anum Zahid, Amr Negm, Ahmed Akrem, Patrick Spencer, Christian Betzel

**Affiliations:** Department of Chemistry, University of Engineering & Technology, G.T. Road, 54890 Lahore, Pakistan; Biochemistry Division, Department of Chemistry, Faculty of Science, Mansoura University, Mansoura, Egypt; Botany Division, Institute of Pure and Applied Biology, Bahauddin Zakariya University, 60800 Multan, Pakistan; Biotechnology Centre, Instituto de Pesquisas Energeticas e Nucleares, Avenida Lineu Prestes, 2242 São Paulo, Brazil; Department of Chemistry, University of Hamburg, 20146, Martin-Luther-King Platz 6, 22607 Hamburg, Germany; Institute of Biochemistry and Molecular Biology, Martin-Luther-King Platz 6, 20146 Hamburg, Germany; Laboratory for Structural Biology of Infection and Inflammation, DESY, Build. 22a Notkestr. 85, 22603 Hamburg, Germany

**Keywords:** Snake venom, Peptides, Angiotensin converting enzyme, Hypotension, MALDI/TOF-TOF, Size exclusion chromatography

## Abstract

**Background:**

Snake venom is a source of many pharmacologically important molecules. *Agkistrodon bilineatus* commonly known as Cantil, is spread over Central America particularly in Mexico and Costa Rica. From the venom of *Agkistrodon bilineatus* we have isolated and characterised six hypotensive peptides, and two bradykinin inhibitor peptides. The IC-50 value of four synthesized peptides was studied, towards angiotensin converting enzyme, in order to study the structure-function relationship of these peptides.

**Results:**

The purification of the peptides was carried out by size exclusion chromatography, followed by reverse phase chromatography. Sequences of all peptides were determined applying MALDI-TOF/TOF mass spectrometry. These hypotensive peptides bear homology to bradykinin potentiating peptides and venom vasodilator peptide. The peptide with m/z 1355.53 (M + H)^+1^, and the corresponding sequence ZQWAQGRAPHPP, we identified for the first time. A precursor protein containing a fragment of this peptide was reported at genome level, (Uniprot ID P68515), in *Bothrops insularis* venom gland. These proline rich hypotensive peptides or bradykinin potentiating peptides are usually present in the venom of Crotalinae, and exhibit specificity in binding to the C domain of somatic angiotensin converting enzyme. Four of these hypotensive peptides, were selected and synthesized to obtain the required quantity to study their IC50 values in complex with the angiotensin converting enzyme. The peptide with the sequence ZLWPRPQIPP displayed the lowest IC50 value of 0.64 μM. The IC50 value of the peptide ZQWAQGRAPHPP was 3.63 μM.

**Conclusion:**

The canonical snake venom BPPs classically display the IPP motif at the C-terminus. Our data suggest that the replacement of the highly conserved hydrophobic isoleucine by histidine does not affect the inhibitory activity, indicating that isoleucine is not mandatory to inhibit the angiotensin converting enzyme. The evaluation of IC 50 values show that the peptide with basic pI value exhibits a lower IC 50 value.

## Background

Snake venom is known for its toxic and lethal effects in its prey. Nature has endowed this creature, with this special secretion to survive in a particular niche [1]. Snake venoms consists of enzymatic and non enzymatic proteinaceous components, which can be grouped into several families based on their structural and functional relationship [2]. Although the members of a single family show remarkable similarities in their primary, secondary and tertiary structures, they often exhibit distinct different pharmacological effects [3]. Studies are being carried not only to unravel and characterize the composition of snake venom, but also to identify and develop novel therapeutics from venoms for the benefit of mankind [3–8]. Particularly venom peptides are today of great interest in this regard, because as a result of the evolutionary process, they have attained highly stable molecular scaffolds, which are resistant to degradation by proteases. Beside these peptides are poorly immunogenic they can be easily synthesized. The structural stability of some venom peptides is a result of disulfide bond formation and posttranslational modification [9]. The most common type of the posttranslational modification observed in snake venom peptides is a pyroglutamate residue at the N-terminus [10]. Bradykinin potentiating peptides (BPP) are a good example in this respect. These peptides were isolated from the venom of *Bothrops jararaca* [11]. Captopril, which was the first orally active inhibitor of the angiotensin converting enzyme was designed based on the structure of BPPs isolated from *Bothhrops jararaca* venom [12]. Since then numerous studies have been made to isolate and characterize BPPs from different snake venoms [13–18]. Efforts are being made to study the structure function relationship of this type of peptides in detail, and their possible modes of blood pressure lowering or vasodilatation. For example it was shown that the peptide Bj-PRO-10c [19] inhibits ACE, however argininosuccinate synthetase, present in the kidney cytosol is its primary target [15, 19–21].

Here we summarize the isolation and characterisation of five bradykinin potentiating peptides, one vasodilator peptide and three bradykinin inhibiting peptides from the venom of *Agkistrodon bilineatus*, generally known as Cantil. In order to determine their IC 50 values, the inhibitory activities of the synthetic analogues of four natural BPPs peptides towards angiotensin converting enzymes were also studied.

## Results

### Isolation of Bradykinin potentiating peptides from *Agkistrodon bilineatus* venom

**Fig. 1:**
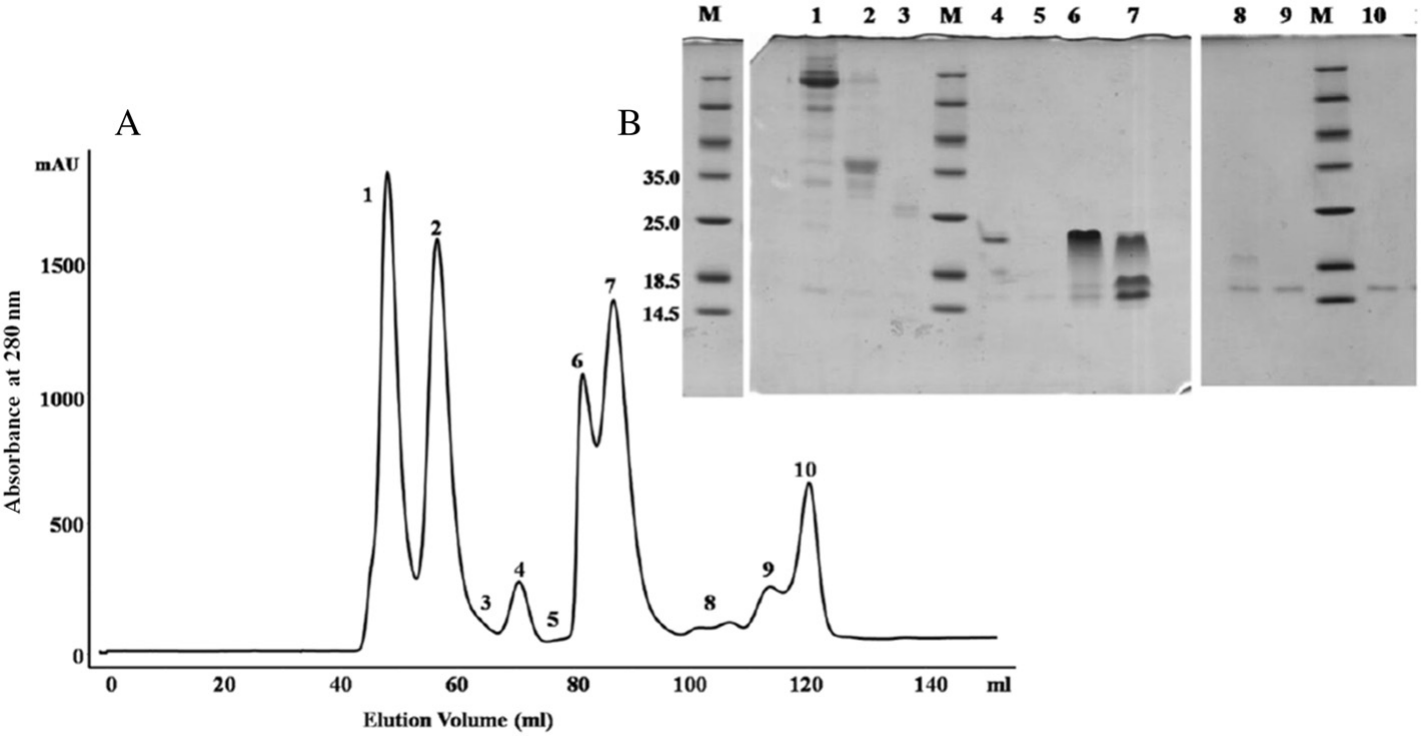
Fractionation of crude *Agkistrodon bilineatus* venom applying a size exclusion column, Superdex-75, 16x60 mm, at pH 5. Inset of the figure shows a SDS-PAGE (15 % glycine, non reducing gel) of the fractions

**Fig. 2:**
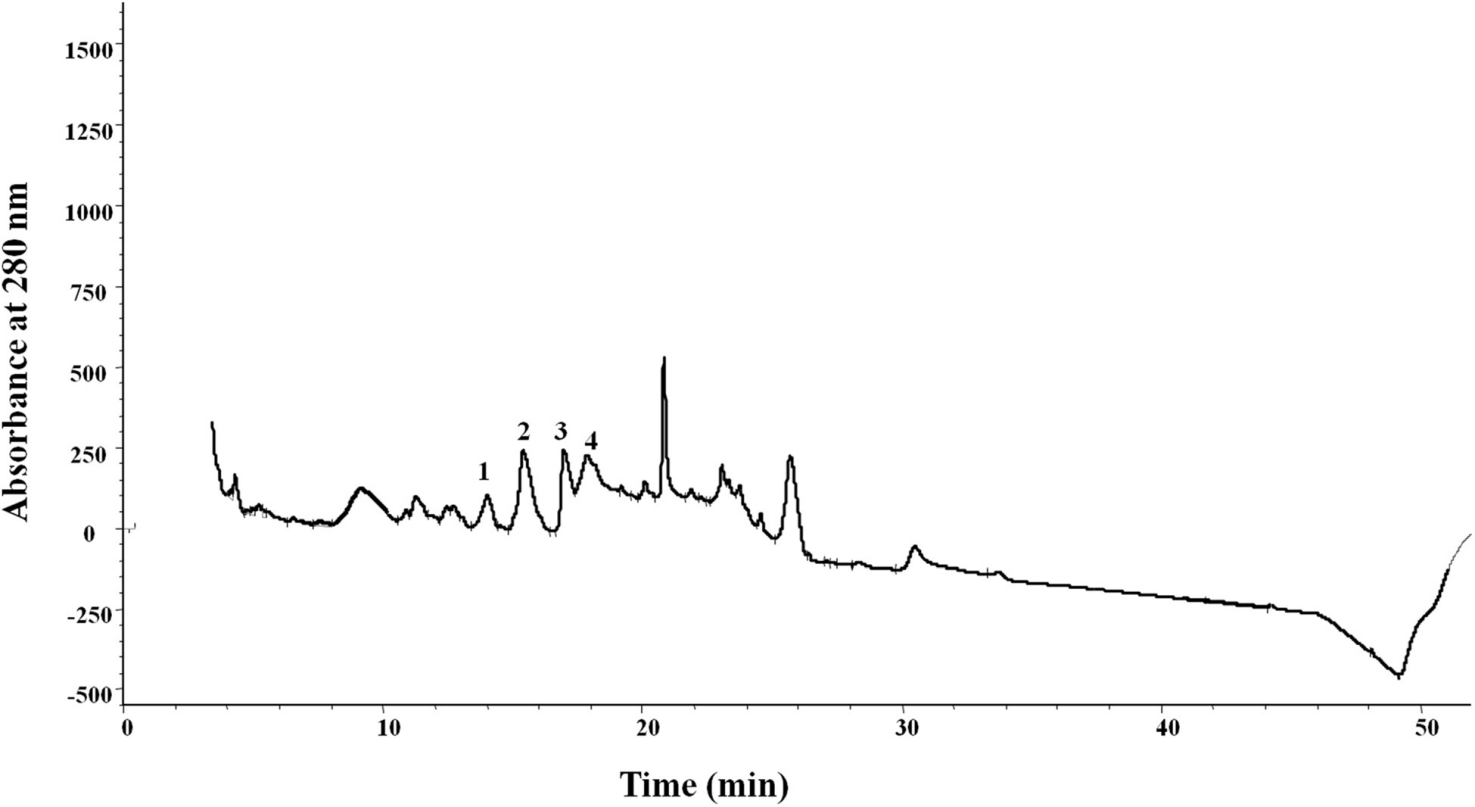
Further purification of peak fractions 8-9, shown before in Fig. 1, after filtering through a 3 kDa membrane, applying a C-18 column (100 X 4.6 mm)

The size exclusion chromatogram (Fig. 1) shows the fractionation of crude *Agkistrodon bilineatus* venom. The inset of the figure shows the SDS-PAGE (Sodium dodecyl sulfate-polyacrylamide gel electrophoresis) of the size exclusion chromatogram peaks, which indicate that the later eluting peaks (from 7 to 10) contains molecules below 18 kDa. The fractions corresponding to Peaks 8 and 9 (Fig. 1) showed inhibition towards the angiotensin converting enzyme. The fractions under these peaks were further fractionated by reverse phase chromatography, on a C-18 column. The activity of the material corresponding to each peak was again tested for inhibitory activity towards the angiotensin converting enzyme and all peptides corresponding to the peaks 1 to 4 (Fig. 2) were found to inhibit the angiotensin converting enzyme. Matrix-assisted laser desorption/ionization time of flight mass spectrometry (MALDI-TOF-MS) demonstrated the presence of peptides between m/z 1019.51- 1370.70 (M + H)^+^. The sequence information of the peptides was obtained by MALDI-TOF/TOF mass spectrometry. Three bradykinin inhibitor peptides were isolated, which differ at their N-terminus. One of these inhibitory peptides has the sequence TPPAGPDVGPR. While the peptide with the molecular mass 1019.51 (M + H)^+^ with the sequence PPAGPDVGPRG lacks a threonine at its N-terminus and has a glycine at its C-terminus, which is not present in the other sequence. In addition to these bradykinin inhibitors six bradykinin potentiating peptides were identified, all of which are homologues to each other. Among these six BPP peptides, five have XPP residues at their C-terminus, a highly conserved motif for BPPs, while the ZQWAQGRAPHPP peptide with 1355.53 Da (M + H)^+^ molecular weight, has a histidine instead of the canonical isoleucine just before the two prolines. Secondly all these BPP peptides have a pyroglutamate, represented by Z (Table 1) at their N-terminus, except the PKVSPRWPPXPP peptide, m/z 1370.70 (M + H)^+^ . This peptide has an atypical N-terminus without glutamate or pyroglutamate.

**Table 1:** Peptides identified in *Agkistrodon bilineatus* venom

RP fraction	Peptide identity	Observed Mass (M + H)^+^	Mascot Score	Sequence	Homology with the peptide	Peptide Family
1	Peptide1	1019.51	18	PPAGPDVGPRG	Q27J49: *Lachesis muta muta*	BPP and CNP
2	Peptide2	1063.54	85	TPPAGPDVGPR	P85025: *Agkistrodon bilineatus*	BPP and CNP
1	Peptide3	1063.54	24	ZSAPGNEAXPP	P0C7K3: *Crotalus viridis viridis*	BPP
3	Peptide5	1196.58	62	ZNWPHPQXPP	Q7T1M3: *Bothrops jararacussu*	BPP and CNP
4	Peptide6	1214.65	19	ZLWPRPQXPP	P0C7S6: *Crotalus atrox*	BPP
3	Peptide7	1355.53	12	ZQWAQGRAPHPP	P68515: *Bothrops insularis*	BPP and CNP
4	Peptide8	1370.70	58	QGGWPRPGPEXPP	P0C7R7: *Bothrops alternatus*	BPP
4	Peptide9	1370.70	21	PKVSPRWPPXPP	P84746: *Bothrops jararacussu*	venom vasodilator peptide

Abbreviations used: Z, pyroglutamate; BPP, Bradykinin potentiating peptide; CNP, C-type natriuretic peptide. X, Isoleucine/Leucine

The peptide sequencing was done in an automated mode, by applying Mascot Inhouse search. Although with MALDI-TOF/TOF, it is not possible to differentiate between isobaric leucine and isoleucine, and quasi-isobaric (Gln/Lys) residues, however previous studies, Edman degradation and cDNA, of similar BPPs, support the presence of the amino acid isoleucine instead of leucine, and Gln rather than Lys [16, 22–24]. Therefore at all places in the manuscript X refers to Ile/Leu.

### Determination of IC 50 values

**Fig. 3:**
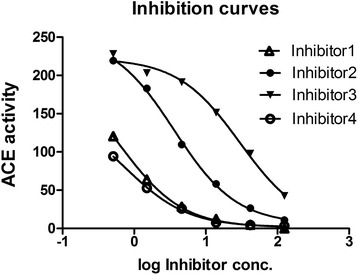
Dose response curves of the synthesized peptides, ACE activity Vs log of inhibitor concentration. The N-terminus of all the synthesized peptides have a pyroglutamate modification, while C-terminus is a free carboxylic acid

The inhibition of ACE by the bradykinin potentiating peptides was observed by measuring the fluorescence of the Dnp group of the ACE fluorescent substrate Abz-Phe-Arg-Lys (Dnp)-Pro-OH. Four BPPs were selected to study the IC 50 values (Table 2). The IC 50 values of these peptides have not been studied before. Peptide 6, with the sequence ZLWPRPQXPP showed the lowest IC 50 value 0.64 μM. The IC 50 value of peptide 7 (ZQWAQGRAPHPP) was 3.63 μM, peptide 5 with a sequence ZSAPGNEAXPP exhibited the highest IC 50 value of 30.00 μM among all the four peptides analysed. Peptide 3 with the sequence ZNWPHPQXPP, had an IC 50 value of 0.76 μM. All four peptides were able to inhibit the ACE activity, but to a different extent. This was also demonstrated by dose response curve (Fig. 3) and the calculation of IC 50 values (Table 2). The IC 50 values of the selected peptide inhibitors reflected a difference in the affinity towards the ACE. The order from stronger to weaker inhibition is found to be as follows: ZLWPRPQXPP > ZNWPHPQXPP > ZQWAQGRAPHPP > ZSAPGNEAXPP. The strongest inhibitors, ZLWPRPQXPP and ZNWPHPQXPP, both have PQXPP at the C terminus.

**Table 2:** IC 50 values of the synthetic analogues of natural BPPs for the angiotensin converting enzyme

Peptide	Sequence	Calculated pI value	IC 50 (μM)
Inhibitor 1 (P6)	ZLWPRPQXPP	9.75	0.64
Inhibitor 2 (P7)	ZQWAQGRAPHPP	9.78	3.63
Inhibitor 3 (P5)	ZSAPGNEAXPP	3.99	30.00
Inhibitor 4 (P3)	ZNWPHPQXPP	6.74	0.76

Z in stands for pyroglutamate; X refers to isoleucine/leucine

## Discussion

**Fig. 4:**
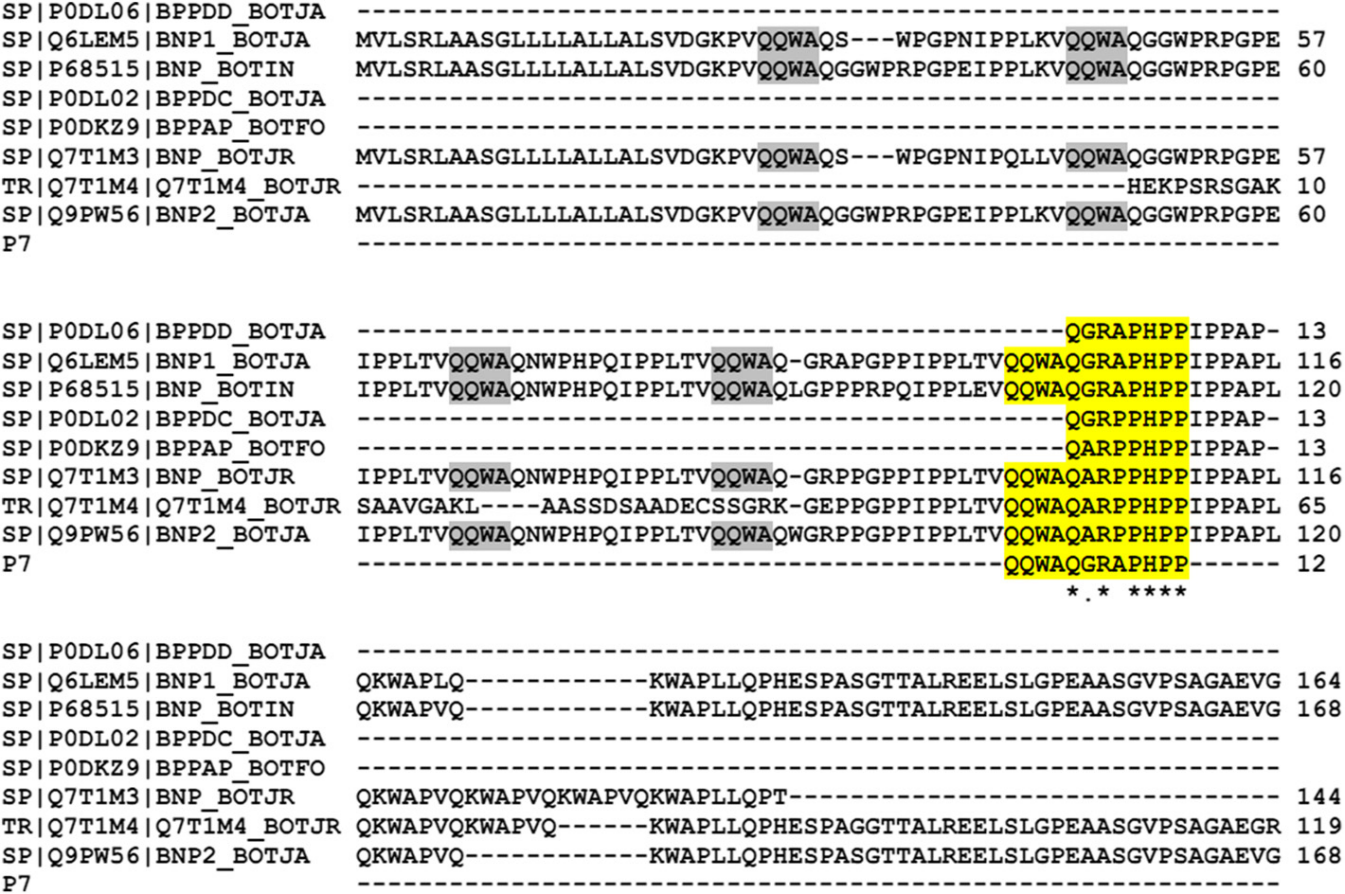
Sequence alignment of peptide 7 (ZQWAQGRAPHPP) with other snake venom BPPs. [13, 14, 28, 29, 31, 40, 41]. Sequence alignment was done with Clustal W

Hypertension is a result of complex interplay of genetic and environmental factors [25], and involves an intricate pathophysiology, with angiotensin converting enzyme playing a key role in the regulation of blood pressure [26]. The first bradykinin potentiating peptide, isolated from the *Bothrops jararaca*, venom became the precursor of today's most commonly used antihypertensive drugs like captopril or lisinopril [11, 12]. After the discovery of the first BPP, homologous of these proline rich peptides have been isolated also from other snake venoms, particularly from the members of Crotalinae subfamily. The BPPs isolated from *Agkistrodon bilineatus* show homology with BPPs isolated from other snake venoms (Table 1). A NCBI data base search showed that the peptide 7 with m/z 1355.53 (M + H)^+^ and a corresponding sequence ZQWAQGRAPHPP was identified for the first time at the peptide level. Previously the sequence of the precursor protein of this peptide was reported at the transcript level in the venoms of *Bothrops insularis* and *Bothrops jararaca* [27, 28]. This peptide lacks the typical IPP motif at its C-terminus, usually present in the snake venom BPPs. However the low IC 50 value (3.63 μM) of this peptide towards ACE suggests that the IPP motif at the C-terminus is not important for the inhibitory activity of snake venom BPPs towards ACE. The presence of a histidine residue next to the two prolines at the C-terminus indicates the potential involvement of this residue in binding to the zinc ion of the angiotensin converting enzyme, thereby inhibiting its activity. Previous studies report the hypotensive effect and ACE inhibitiory ability, of similar BPPs by employing classical pharmacological assays or studying inhibition constants of BPPs over ACE. Since various methods were used for the assay, it is difficult to compare inhibitory ability of BPPs, isolated in this study, with other BPPs having different sequences. Ki value of Bj-PRO-10C was recently reported to be 0.20 μM [24]. BPP-XIe had a Ki value of 0.084 μM and that of BPP-AP was 0.035 μM [18]. The sequence alignment (Fig. 4) shows the homology of peptide 7 with other BPP and CNP type peptides. The same residues are highlighted in yellow colour. It can be inferred from Fig. 4, that in addition to lacking a usual IPP sequence at the C-terminus, peptide 7 also has a unique N-terminus, having a sequence QQWA. The sequence QQWA, which has been highlighted in grey colour, appears several times in the precursor, always before a mature BPP. The sequence analysis of the precursor proteins and the mature peptides (Fig. 4) [14, 27, 29–31], gives a clue that QQWA must be a cleavage site, that might have been misinterpreted by the processing mechanism along the maturation of peptide 7. Also the missing IPP C-terminus in peptide 7 (ZQWAQGRAPHPP) is exactly after HPP on the precursor sequence (P68515, Fig. 4) indicating that the novel peptide 7 is a different processing product of a canonical BPP precursor.

**Fig. 5:**
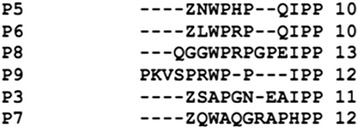
Sequence alignment of BPPs isolated and characterized in the present work, in order to study points of similarities and difference. The alignment was done with Clustal W

A clustal w sequence alignment of the BPPs isolated in this work is shown in Fig. 5. All these peptides have two prolines at their C-terminus, and the preceding residue in all peptides is an isoleucine, except in peptide 7, which has a histidine at this position. Four peptides (P3, P5, P6, P7) have a pyroglutamate at their N-terminus, while P8 has a glutamine and P9 has a proline instead. The presence of pyroglutamate modification at the N-teminus indicates the natural resistance to degradation to these type of peptides. Studies have shown that the presence of PP residues at the C -terminus of the peptides confers resistance to hydrolysis by peptidases [32]. The analysis of crystal structure of Ang II in complex with sACE, showed that the proline, which is the seventh residue of Ang II, prevents hydrolysis of the peptide by ACE, thus inhibits it [33], and hence could serve as a negative feedback mechanism in the regulation of Ang II. Further the study of crystal complex of C-domain of somatic s-ACE and BPPb showed that the two C-terminal prolines of the peptide strongly interacts with the primary binding site, through the formation of hydrogen bonds with multiple residues [33]. Therefore the presence of several proline residues in BPPs, has equipped these peptides with inherent resistance to hydrolysis. The data obtained in the present work and literature studies show that isoleucine is usually present next to the two C terminus prolines [14–16, 18, 20, 34–36]. The results of the IC 50 values obtained for the synthesized peptides (Table 2) illustrate that while peptides 5, 6 and 7 show low IC 50 values, peptide 3 has a much higher value. As shown in Table 2 peptide 3 has the lowest pI value among all the four synthesized peptides. Further, sequence analysis of these four peptides (Fig. 5) shows that in peptide 3 two polar residues asparagine and glutamic acid are present next to each other, while in the other three peptides the charged amino acid residues are flanked by either proline, glycine, or tryptophan. The low pI value and the presence of the two charged residues together, might be responsible for the high IC 50 value of this peptide, as these factors might hinder the appropriate binding of this peptide within the active site of ACE. In addition to these BPPs, three bradykinin inhibitory peptides were also isolated and characterized. Peptide 2 was previously reported by Graham, *et. al* [37] in *Agkistrodon bilineatus* venom, indicating that these peptides have been preserved as an essential component of snake venoms*,* over the process of evolution. The same peptide 2 was recently reported from the venom of *Agkistrodon bilineatus howardgloydi* [38]. A systematic study of BPPs, in terms of their sequence, physical properties and molecular targets might lead to a complete understanding of the mechanism of hypertension induced by these peptides, and will support the design of new drugs with more efficacy, specificity and lower side effects.

## Conclusion

Snake venoms, are a source of many molecules with biomedical importance. The BPPs isolated and characterized within this work show homology with BPPs already isolated from other snake venoms. However, an extensive data base search revealed that peptide 7 with the sequence ZQWAQGRAPHPP has been identified for the first time. All previous descriptions of this peptide were inferred from transcripts, with no evidence of secretion of the gene product in the venom. The analysis of IC 50 values (Table 2) for ACE, applying four synthetic replica of these BPPs, provided further insights into the structure-function relationship of these peptides. The low IC 50 value of the synthetic peptide ZQWAQGRAPHPP indicates that the presence isoleucine in the IPP sequence is not mandatory to inhibit ACE. Furthermore the calculated pI values of these peptides suggest that the peptides with basic pI value show a lower IC 50 value. Secondly the sequence analysis of the BPPs isolated and described in this work and other studies [13, 14, 18, 34], show that these peptides usually have a pyroglumate modification at their N-terminus, and have a high ratio of proline residues. These two factors provide inherent stability to these peptides towards degradation, particularly in venom glands, which contain various types of enzymes, as well as in the prey's blood. Further, it can be concluded that the presence of peptide 2 (bradykinin inhibitor peptide) reinforces the idea that these peptides are conserved in the venom of *Agkistrodon bilineatus* over the process of evolution and that these molecules, so far found in many venoms, must play a key role in predation.

## Experimental

### Materials

The snake venom of *Agkistrodon bilineatus bilineatus*, was obtained from Venom Supplies, Australia, and was placed at -20° till further use. The venom was collected from the snakes bred in the farms of Venom Supplies, Australia. All the solvents used were of HPLC grade and were obtained from Merck. The substrate Abz-Phe-Arg-Lys (Dnp)-Pro-OH for angiotensin converting enzyme was obtained from BACHEM. Angiotensin converting enzyme from rabbit lung (A6778- 0.25 UN), was obtained from Sigma. The synthesized peptides ZLWPRPQIPP, ZQWAQGRAPHPP, ZSAPGNEAIPP, ZNWPHPQIPP were purchased from China Peptide company, Shanghai, China. The peptides were 95 % pure, and were kept at -20° till use. The N-terminus of these synthetic peptide is pyroglutame (represented by Z), while the C-terminus is an amino acid with free carboxylic group.

## Methods

### Purification of Bradykinin potentiating peptides (BPP)

The crude venom (50 mg) was dissolved in 1 ml of 100 mM ammonium acetate (pH 5), and centrifuged at 13000 rps. The undissolved material settled down, and the supernatant of crude venom solution was fractionated using a size exclusion column (Superdex-75, 16 x 60 mm) connected to an ÄKTA Purifier system (GE Healthcare). 100 mM ammonium acetate (pH 5) was used as the elution buffer. The fractionation was performed at a rate of 1 ml/min, and UV absorbance of the eluate was monitored at 220 and 280 nm. Fractions were collected and subjected to SDS-PAGE (15 % glycine gels) under non reducing conditions. The gels were stained with Coomassie Blue. The peptide fractions (peak 8-9, Fig. 1) showing inhibitory activity towards the angiotensin converting enzyme were filtered through 3 kDa Amicon filter and further purified with Chromolith-C-18 column (100x 4.6 mm), using an Agilent 1200 system. Solvent A was 0.2 % formic acid in water and B was straight acetonitrile. A stepwise gradient 3-30 % B for 28 min, 30-40 % B for 9 min and 40-60 % B for 3 min and a flow rate of 2 ml/min was applied to isolate the peptides (Fig. 2). UV absorbance was monitored at 220 nm and 280 nm.

### Enzyme inhibition assay

ACE activities in the presence of venom peptides were determined by a fluorescence energy transfer assay using Abz-Phe-Arg-Lys (Dnp)-Pro-OH as a substrate [39]. 1 mg of the substrate was weighed and dissolved in 1 ml DMSO. The exact concentration of the substrate was determined by taking four different volumes of the substrate stock solution, and constructing a standard curve spectrophometrically at 365 nm, using the molar extinction coefficient of the Dnp group (ε_356_ = 17,300 M^−1^ cm^−1^), according to the Beer’s Lambert Law (A = ε_Dnp_ x l x c). A stock solution of the enzyme was prepared by suspending 0.25 UN of ACE in 250 μl of the assay buffer (12.10 g Tris-base, 2.92 g NaCl and 1.36 mg ZnCl_2_ in 1 l of deionised water, pH to 7.0 adjusted with HCl).

In order to determine the IC 50 values of the synthesized peptides, a stock solution (10 mM) of each was prepared in the assay buffer. From the stock solutions, six serial dilutions were prepared for each peptide. The concentrations of the six dilutions were as follows, 0.500 mM, 0.167 mM, 0.056 mM, 0.019 mM, 0.006 mM and 0.002 mM respectively. In the final assay, 85 μl of the assay buffer, 2 μl of the enzyme stock solution, and 5 μl of the dilute peptide solution were added. The mixture was incubated for 10 min at room temperature. The reaction was started by adding 10 μl of the working solution of the substrate (20 μl of the stock +280 μl of the assay buffer). The same procedure was adopted using 5 μl of venom fractions instead of the synthetic peptides. Fluorescence measurements were made at λ_ex_ = 320 nm and at λ_em_ = 420 nm, for 5 min each. These experiments were repeated five times to ensure accuracy of the results.

### Matrix-assisted desorption/ionization time-of-flight mass spectrometry

MALDI-TOF-TOF analyses were performed with a ultrafleXtreme instrument (Bruker Daltonics, Bremen, Germany). Samples were dried after reversed phase chromatography, dissolved in 30 % ACN, 0.1 % TFA in H2O and 1 μl of the solution was spotted on a MALDI target plate (MTP AnchorChip 384, Bruker Daltonics). After drying 1 μl MALDI matrix (0.7 mg/ml Cyano-4-hydroxycinnamic acid (Bruker Daltonics) dissolved in 85 % ACN, 1 mM NH4H2PO4 and 0.1 % TFA dissolved in H2O) were spotted on the sample plate.

Data acquisition was performed in positive ion mode using the flexControl software 3.3. The parameters were set as follows: ion source 1: 25 kV, ion source 2: 23.6 kV, lens: 7.5 kV. MS data were collected automatically using autoXecute. Parameters were set as follows: laser power: 47 %; laser shots: 1000; movement, random walk with 100 shots per raster spot. Peaks were selected for LIFT measurement if they met the following criteria: signal to noise > 8, peak intensity > 300.

MS spectra were processed applying flexAnalysis (version 3.3, Bruker Daltonics). Further data analysis was performed using BioTools (version3.2, Bruker Daltonics) and Mascot Inhouse Search. Mascot version 2.1.03 was used to analyse and search the spectra against the subset “other lobe-finned fish and tetrapod clade” of the Swissprot database. The precursor ion mass tolerance was set to 1 Da, the fragment ion mass tolerance was 0.5 Da.

**Fig. 6:**
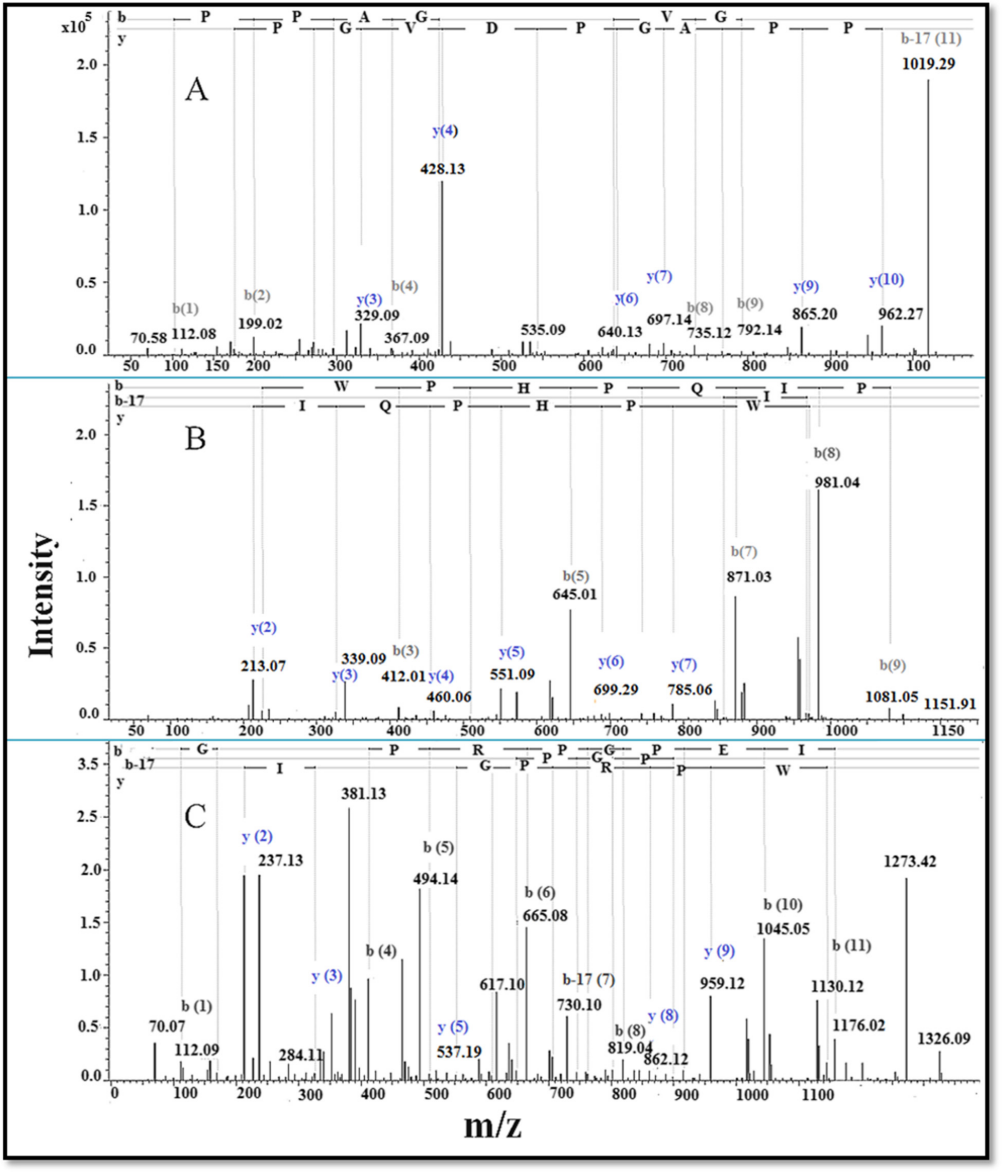
MALDI-TOF/TOF-MS of selected peptides. **a**. TPPAGPDVGPR [m/z 1063.55 (M + H)^+^]; **b**. ZNWPHPQIPP [m/z 1196.58 (M + H)^+^]; **c**. QGGWPRPGPEIPP [m/z 1370.70 (M + H)^+^]. The figures show b and y ion series and the sequence annotation of the corresponding peptide

The sequence annotation pictures of the representative peptides, prepared applying the Bruker software ProteinScape, version 3.0, are shown in Fig. 6.

## Abbreviations

ACE: angiotensin converting enzyme
BPP: bradykinin potentiating peptides
Z: pyroglutamate
X: Isoleucine/leucine
